# Sodium Dodecyl Sulphate-Supported Nanocomposite as Drug Carrier System for Controlled Delivery of Ondansetron

**DOI:** 10.3390/ijerph15030414

**Published:** 2018-02-27

**Authors:** Gaurav Sharma, Mu. Naushad, Bharti Thakur, Amit Kumar, Poonam Negi, Reena Saini, Anterpreet Chahal, Ashok Kumar, Florian J. Stadler, U.M.H. Aqil

**Affiliations:** 1 Shenzhen Key Laboratory of Polymer Science and Technology, Guangdong Research Center for Interfacial Engineering of Functional Materials, Nanshan District Key Lab for Biopolymers and Safety Evaluation, College of Materials Science and Engineering, Shenzhen University, Shenzhen 518060, China; mittuchem83@gmail.com (A.K.); fjstadler@szu.edu.cn (F.J.S.); 2 Key Laboratory of Optoelectronic Devices and Systems of Ministry of Education and Guangdong Province, College of Optoelectronic Engineering, Shenzhen University, Shenzhen 518060, China; 3 School of Chemistry, Shoolini University, Solan 173212, India; bt3456thakur@gmail.com; 4 Department of Chemistry, College of Science, Bld.#5, King Saud University, Riyadh 11451, Saudi Arabia; 5 School of Pharmaceutical Sciences, Shoolini University, Solan 173212, India; poonamgarge@gmail.com; 6 School of Applied Science and Biotechnology, Shoolini University, Solan 173212, India; reenavohra10@gmail.com (R.S.); chahal2407@gmail.com (A.C.); 7 Department of Biotechnology and Bioinformatics, Jaypee University of Information Technology, Waknaghat, Solan 173234, India; ashok.nadda09@gmail.com; 8 School of Public Health, 3rd Floor, SRM Medical College and Research, Centre, Kattankulathur 603211, India; haideraqil@gmail.com

**Keywords:** sodium dodecyl sulphate, nanocomposite, drug delivery, ondansetron

## Abstract

Sodium dodecyl sulphate-supported iron silicophosphate (SDS/FeSP) nanocomposite was successfully fabricated by the co-precipitation method. The SDS/FeSP nanocomposite was investigated as a drug carrier for ondansetron. The cumulative drug release of ondansetron was observed at various pH values for different time intervals, i.e., from 20 min to 48 h. A ranking of the drug release was observed at different pHs; pH 2.2 > saline (pH 5.5) > pH 7.4 > pH 9.4 > distilled water. Maximum release of encapsulated drug was found to be about 45.38% at pH 2.2. The cell viability tests of SDS/FeSP nanocomposite concluded that SDS/FeSP nanocomposite was non-cytotoxic in nature.

## 1. Introduction

In the modern era, nanoparticles are being explored in many areas such as drug delivery, bio-imaging, therapeutics and other diagnostic applications. Because of their defined nanosize and unique surface compositions, they can act as desirable carriers for controlled drug delivery systems. Widely used nanoparticles include metals, carbon nanotubes, quantum dots, ceramic and magnetic nanoparticles, etc. [1,2]. Nanoparticles, especially polymeric and mesoporous silica nanoparticles, have been investigated widely [3,4]. In the field of biomedicine, metallic nanoparticles such as silver, gold, cobalt, platinum, iron, nickel, etc. are among the most investigated. Metallic nanoparticles are generally more stable than polymeric and biological nanoparticles [5]. Thus metallic nanoparticles can be used for diverse applications owing to their distinctive properties [6], but their large surface area and small size can lead to particle-particle agglomeration which makes their physical handling difficult for nanoparticles in liquid and dry forms. The small size particles and large surface area easily result in limited drug loading and burst release. Such practical issues have to be overcome before nanoparticles are to be utilized clinically or made commercially available. Agglomeration of nanoparticles can be prevented by the use of suitable coatings. Several such coatings which are used to prevent agglomeration are poly(vinylpyrrolidone) (PVP), polyethylene glycol (PEG), natural polymers (dextran, guar gum, chitosan, etc.) and surfactants, etc. [7,8,9]. Attempts have been made to introduce functionality and improve the characteristic properties of nanoparticles, such as use of functionalized acid dopants during their synthesis and copolymerization with an organic matrix for the formation of nanocomposites, etc. [10,11,12].

Significant development in this area is attributed to composite metal nanoparticles. They show properties corresponding to the combination of two materials of different nature and the obtained materials have wider performance characteristics of the individual components taken separately. Nanocomposites consist of one or more discontinuous phases distributed in one continuous phase. The continuous phase is called matrix and the discontinuous phase is called the reinforcing material. To enhance the functional properties of organic materials, inorganic materials such as metals and metal oxides are often incorporated to form multi-functionalized composites for various applications in the fields of energy, electronics, sensors, catalysis and biomedicine, etc. [13,14]. Iron-based nanocomposites have received attention as drug carriers due to their enhanced loading capabilities and control over their physicochemical properties. Such nanocomposites not only possess ferromagnetic properties but also display the properties of organic compound which are non-toxic and suitable for biology and medicine applications [2,15,16]. These composites can be synthesized by various methods, e.g., sol-gel, chemical precipitation, hydrothermal, micro-emulsion precipitation, surfactant mediated precipitation and electrodeposition, etc. These processes require the choice of suitable pH, temperature, concentration of reactants, method of mixing and rate of oxidation, etc. To reduce high chemical activity and oxidizing power of metallic nanoparticles, grafting or coating with organic species (polymers/surfactants) or inorganic layers (carbon/silica) are used. These techniques are utilized to confer advanced functions to the surface of polymers which provide effective interactions between the drug delivery system and the therapeutic drug so that the latter may be used in chemotherapy causing minimum adverse effects [17].

Surfactant-based drug delivery systems typically have reduced side effects and therefore better patient compliance. The unique features of the composites are the presence of optimal proportions of hydrophobic and hydrophilic parts and the number of free hydroxyl groups in the molecule. Surfactants have shown good properties for drug delivery systems and thus can compete with synthetic biodegradable materials which are safe, economic and non-toxic [18]. Such nanocarriers can provide versatile platforms for the delivery of many pharmacological agents, particularly to enhance the therapeutic effect and overcome drug resistance. The biodegradable surfactants-based nanocomposites have the properties of degrading in biological fluids with progressive release of the dissolved drug. Numerous surfactant-based novel drug delivery approaches have been developed. Biosafety and biocompatibility are the important characteristics required for the use of surfactant-based nanocomposites in the field of pharmaceutical formulation and drug delivery. Biodegradable surfactant-based nanocomposites find widespread use in drug delivery as they can be degraded to non-toxic monomer units inside the body [19].

The aim of this work was to fabricate a novel material for controlled release of ondansetron to provide prolong gastric retention and increase the efficacy of the dosage form [20]. The prolonged gastric retention enhances bioavailability, solubility of the drug at high pH and reduces drug waste. It is well known that gastric residence time (GRT) is one of the significant factors which affect the drug bioavailability of pharmaceutical dosage forms. A short gastric emptying time results in incomplete drug release from the drug delivery system [21]. The oral drug administration route is the most desirable route due to the ease of administration, patient compliance, immediate release, site specificity and flexibility in the formulations. In this way the drug is mainly absorbed in the stomach and removed from the body by the liver and kidney [22,23,24]. Keeping in view the above facts in this work a surfactant-based iron silicophosphate nanocomposite has been synthesized, characterized and studied as a drug carrier for the controlled release of ondansetron.

## 2. Materials and Methods

### 2.1. Reagents and Instruments

The reagents used in the study were ferric nitrate (CDH Pvt. Ltd., Delhi, India), sodium silicate (CDH Pvt. Ltd.), orthophosphoric acid (Qualigens, Mumbai, India), sodium dodecyl sulphate (CDH Pvt. Ltd.), nitric acid (CDH Pvt. Ltd.), potassium chloride (CDH Pvt. Ltd.), sodium nitrate (CDH Pvt. Ltd.), the drug ondansetron (Meridian Medicare Pvt. Ltd., Solan, India). A digital pH meter (Elico L1-10, Pune, India), UV–visible spectrophotometer (Systronics 2202, Gujarat, India), Fourier transform infrared (FTIR) spectrophotometer (Perkin Elmer, Hopkinton, MA, USA), scanning electron microscope (SEM Qant-250, model 9393, ThermoFisher Scientific, Hillsboro, OR, USA) and transmission electron microscope (TEM Tecnai G^2^ 20 S-Twin, FEI company, Hillsboro, OR, USA) were used. Dynamic light scattering (DLS) was performed on a Zetasizer Nano (DTS ver.4.10, Serial no. MA L 500962, Malvern Instrument, Malvern, UK).

### 2.2. Synthesis of Sodiumdodecylsulphate Ironsilicophosphate Nanocomposite (SDS@FeSP)

The sodium dodecyl sulphate iron silicophosphate nanocomposite was prepared by adding sodium silicate, orthophosphoric acid and sodium dodecyl sulphate into ferric nitrate at a volume/volume ratio of 1:1:1:1 with constant stirring on a magnetic stirrer at 60 °C for 1 h [25]. Then the pH was adjusted to between 0 and 1 by adding nitric acid. The resultant light brown coloured precipitates obtained were kept for digestion for 24 h with the mother liquor. The supernatant was poured off and the precipitates were filtered and washed with distilled water. The material obtained was dried in an oven at 40 °C. The dried material was cracked into small size granules. The material was then treated with 0.1 M HNO_3_ for 24 h to convert it into the H^+^ form. The ion exchange capacity of the synthesized materials was investigated by a standard column process [26,27,28]. 

### 2.3. Characterization Techniques

FTIR spectra of SDS/FeSP and ondansetron-loaded SDS/FeSP were obtained using the KBr disc method. The materials were mixed with KBr, powdered and discs were formed by applying pressure. FTIR spectra were recorded in the region between 400 and 4000 cm^−1^. Morphological studies of SDS/FeSP nanocomposite were attempted by using SEM. Microphotographs of the nanocomposite were recorded using a high energy electron beam. The interaction of these electrons with the atoms of the sample produce signals which shows the surface morphology of the synthesized nanocomposite. TEM provide information about the particle size of the material. The material was suspended into ethanol and placed over a carbon copper grid. TEM provides high resolution pictures of the material and provides information about its topographical and morphological aspects. Dynamic light scattering (DLS) particle size distribution of the SDS/FeSP nanocomposite was performed after 1 h of ultrasonication of the sample at 25 °C.

### 2.4. Measurement of Zeta Potential

The zeta potential of the nanocomposite was used for studying the electrokinetic potential in colloidal dispersion. The zeta potential indicates the stability of colloidal dispersions. The measurement was obtained at 1.33 (nm) position, at a room temperature of 25 °C in a disposable sizing cuvette. The scattering intensity data were analysed by a digital co-relator under unimodal analysis mode. The dispersion was diluted with water to ensure the light scattering signal capture, as indicated by the particle counts per seconds.

### 2.5. Drug Delivery

#### 2.5.1. Stock Solution

A standard stock solution of ondansetron was prepared by dissolving 20 mg of drug in 500 mL of double distilled water to give a concentration of 40 μg/mL.

#### 2.5.2. Determination of λ_max_

The standard solution of ondansetron was scanned in the wavelength region of 200–400 nm. The λ_max_ for ondansetron was found to be at 248 nm.

#### 2.5.3. Preparation of Ondansetron Standard Calibration Curves

An appropriate aliquot was pipetted from a standard stock solution into a series of 20 mL volumetric flasks to make solutions of varying concentrations, i.e., 2, 4, 6, 8, 10, 12, 14, 16, 18, and 20 µg/mL. The volume was made up to the mark with double distilled water, pH 2.2 acetate buffer, pH 7.4 or pH 9.4 phosphate buffers and saline (pH 5.5) solutions. The absorbance versus concentration were plotted to get standard calibration curves for the different solutions as mentioned above. The concentration of drug was determined by taking λ_max_ on UV-Vis spectrophotometer.

#### 2.5.4. Ondansetron Loading on SDS-FeSP

Loading of ondansetron on the SDS-FeSP nanocomposite were carried out by immersing 100 mg of nanocomposite material in 100 mL of 100 µg solution for 1, 2, 3, 4, 5, 6 and 7 h. The concentration of loaded drug was determined by taking supernatant solution and recording its absorbance values by using the UV spectrophotometer. The drug loading efficiency was calculated by following equation [29,30]:

(1) $$\mathrm{DLE}\;\left(\%\right)=\frac{\mathrm{Total}\;\mathrm{amount}\;\mathrm{ofdrug}-\mathrm{amount}\;\mathrm{offree}\;\mathrm{drug}}{\mathrm{Total}\;\mathrm{mass}\;\mathrm{of}\;\mathrm{composite}}\;\times 100$$

#### 2.5.5. Drug Release at Different Physiological Conditions

The drug release was studied in simulated GIT fluids conditions (pH 2.2, saline, pH 7.4 and pH 9.4) and in distilled water. A sample (100 mg) was added to 100 mL buffer solutions of pH 2.2, pH 7.4, pH 9.4, saline and distilled water [31]. Test samples (2 mL) were withdrawn at time intervals of 20, 40, 60, 120, 180, 240, 300, 360, 420, 480, 720, 1440 and 2880 min. The absorbance value was observed by using the UV spectrophotometer. The % drug release was calculated using the following equation [32]:

(2) $$\mathrm{Drug}\;\mathrm{release}\;\left(\%\right)=\frac{C_{t}}{C_{o}}\times 100$$

where C_o._ and C_t_ are the amount of drug loaded and amount of drug released at time ‘t’, respectively.

#### 2.5.6. Cumulative Drug Release

The cumulative drug release of ondansetron was studied for SDS/FeSP nanocomposite at different physiological conditions and is calculated by using the following equation:

(3) Cumulative drug release (%)=volume of sample withdrawn×P(t−1)×Pt

where, P(t − 1) is % release previous to “t” and Pt is % release at time “t”.

### 2.6. Cytotoxicity Studies

#### 2.6.1. Preparation and Cultivation of PBMC

Blood was obtained from healthy human donors. Informed consent was obtained from all donors and this work was carried out in compliance with the ethical committee guidelines of Shoolini University, Solan. Peripheral blood mononuclear cells (PBMCs) were isolated from the blood using a Ficoll-Paque Plus system according the instructions of the manufacturer. The blood sample was diluted with the same volume of PBS. Afterward, the diluted blood sample was carefully layered on the Ficoll-Paque Plus (Amersham Biosciences, Piscataway, NJ, USA). The mixture was centrifuged at 400× *g* for 40 min at 18–20 °C. The undisturbed lymphocyte layer was cautiously transferred out. The lymphocyte was washed and pelleted down with three volumes of PBS twice and resuspended in RPMI-1640 media supplied with 10% fetal bovine serum (FBS), 2 mM l-glutamine and antibiotics (100 µg/mL penicillin and streptomycin). Cell counting was accomplished to determine the PBMC cell number with an equal volume of trypan blue [33].

#### 2.6.2. Cell Viability and Cytotoxicity Assay

The effect of the SDS-FeSP nanocomposite on cell cytotoxicity was assessed by a MTT assay [34,35]. MTT is a pale yellow substance reduced by living cells to form a dark blue formazan product. This process needs active mitochondria, and even fresh dead cells do not reduce significant amounts of MTT. PBMC (5 × 10^5^ cells/mL) treated with SDS-FeSP nanocomposite was seeded in 10% RPMI-1640 medium in a 96-well plate. Four different concentrations of each sample were taken, i.e., 25, 50, 100 and 200 µg/mL. Concanavalin A-treated PBMC and DMSO-treated PBMC were used as positive and negative controls, respectively. The 96-well plate was incubated in 37 °C, 5% CO_2_ for 48 h. After incubation, the medium was replaced with fresh 10% RPMI-1640 medium containing SDS/FeSP nanocomposite. For MTT assay, 10 μL of MTT (5 mg/mL) was added into each well to generate formazan, and then cells were incubated in humidified atmosphere with 5% CO_2_ at 37 °C for 4 h. After eliminating the supernatant, 100 μL DMSO was added to dissolve the purple crystals with 10 min. incubation. The optical density of each well was measured at 595 nm by a microplate reader. Each extract and control was assayed in triplicate for three times. The percentage proliferation was found by the following formula:

(4) $$\%\;\mathrm{Proliferation}=\frac{\left(O.D.\mathrm{sample}-O.D.\mathrm{control}\right)}{O.D.\mathrm{control}}\times 100$$

## 3. Results and Discussion

The sodium dodecyl sulphate iron silicophosphate nanocomposite was prepared by a simple co-precipitation method. The SDS/FeSP showed an ion exchange capacity, of 1.03 meq g^−1^ for K^+^ ions. The addition of surfactant provided mechanical strength and large surface area.

### 3.1. Characterization

**Figure 1:**
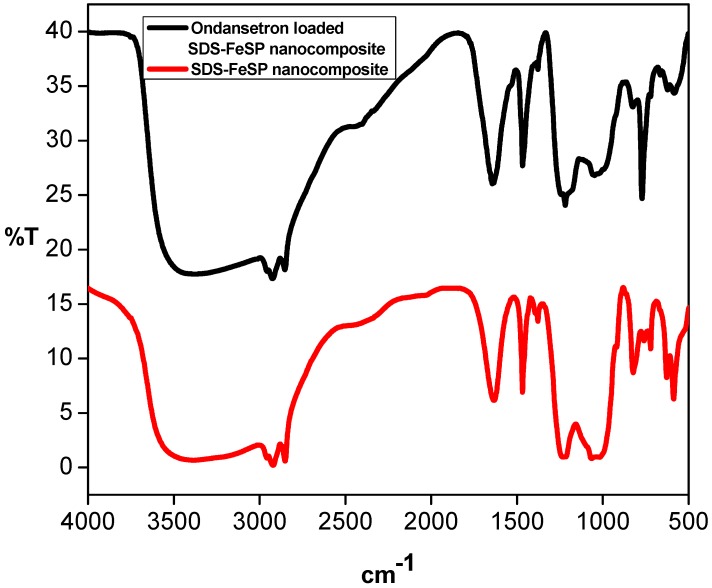
FTIR spectra of ondestron-loaded SDS-FeSP and SDS-FeSP nanocomposite [25].

The FTIR spectra of SDS/FeSP and ondansetron-loaded SDS/FeSP are shown in Figure 1. The peaks at 593 cm^−1^ and 576 cm^−1^ indicate the presence of iron in SDS/FeSP and ondansetron-loaded SDS/FeSP [36,37]. The peaks at 1081 cm^−1^ and 1041 cm^−1^ show the presence of phosphate groups [38]. The intense peaks at 723 cm^−1^, 792 cm^−1^, and 1230 cm^−1^ might be due to the metal-oxygen, metal-hydroxide bonding and sulphate groups of the SDS-FeSP nanocomposite. The peak observed at 1470 cm^−1^ is related to CH_2_ wagging. The peaks at 3393 cm^−1^ and 3432 cm^−1^ correspond to –OH groups, whereas asymmetric C–H stretching vibration produces the peak at 2922 cm^−1^ [39]. 

The presence of atmospheric CO_2_ can be seen in the peak at 1383 cm^−1^. The FTIR spectrum of the nanocomposite with ondansetron showed a characteristic peak at 1644 cm^−1^ which confirms the presence of the C=N in an aromatic ring. The peak at 1219 cm^−1^ is assigned to the C–C stretching in the aromatic ring of the drug.

**Figure 2:**
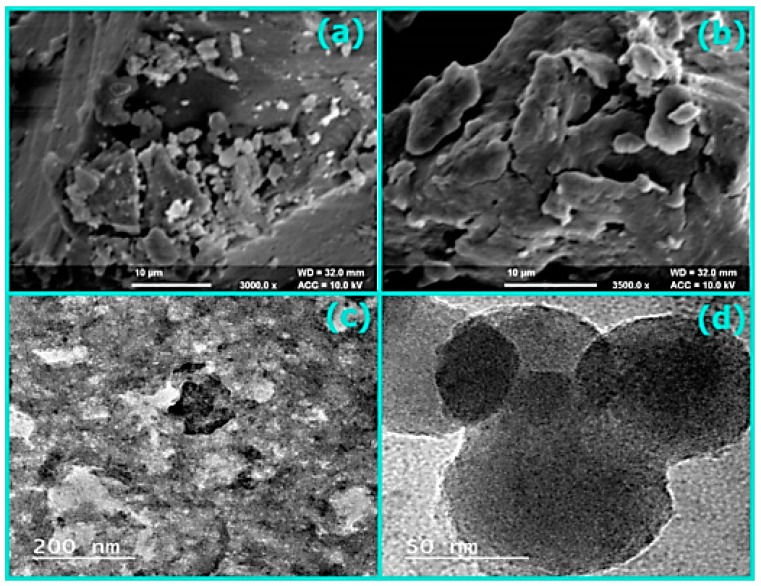
SEM images (**a**) FeSP and (**b**) SDS/FeSP, TEM images of (**c**,**d**) SDS/FeSP nanocomposite [25].

SEM images of SDS/FeSP nanocomposite at different magnifications are shown in Figure 2a,b. The surface morphology clearly showed the fibrous and granular appearance with smooth edges of SDS/FeSP nanocomposite. TEM images of SDS/FeSP nanocomposite at different magnifications are shown in Figure 2c,d. The TEM images indicated the smooth surface appearance of the SDS-FeSP nanocomposite.

**Figure 3:**
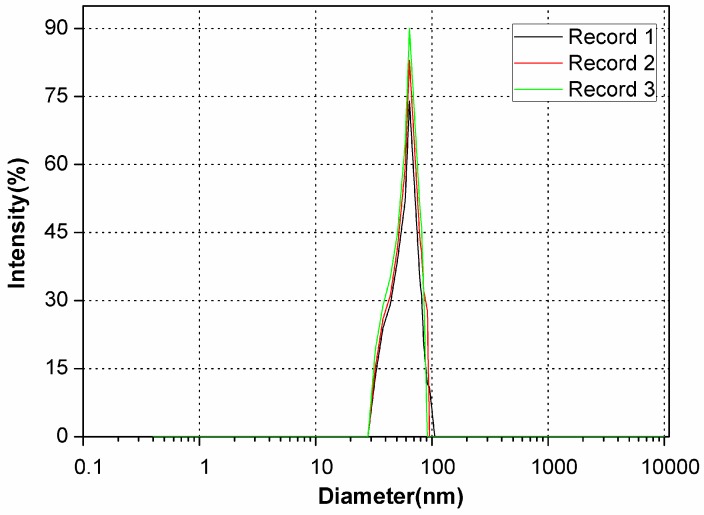
Dynamic light scattering (DLS) particle size distribution of SDS/FeSP nanocomposite. The measurements were performed of the samples after 1 h of ultrasoncation at 25 °C.

The TEM micrographs of SDS/FeSP confirmed that average sizes of the particles were in the range of 30–90 nm. Thus SDS/FeSP material is a nanocomposite. As shown in Figure 3 the SDS/FeSP had average diameters of 63 ± 2 nm observed by DLS and particle size ranges from 30 to 90 nm which is in agreement with the TEM data.

### 3.2. Measurement of Zeta Potential

**Figure 4:**
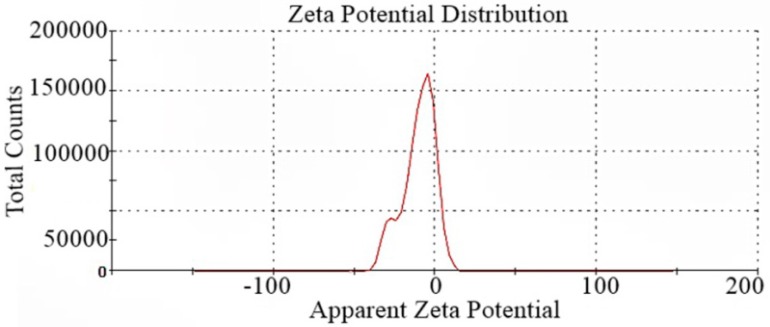
Zeta Potential for SDS/FeSP nanocomposite.

The zeta potential result of the SDS-FeSP nanocomposite is shown in Figure 4, which indicates the stability of the colloidal dispersions. The magnitude of zeta potential, i.e., −10.3 mV, indicates that the SDS/FeSP nanocomposite shows incipient instability with a low rate of coagulation or flocculation [40]. The negative value obtained for the zeta potential indicates that the SDS-FeSP nanocomposite surface is negatively charged.

### 3.3. Drug Delivery

#### 3.3.1. Standard Calibration Curves

Standard calibration curves of ondansetron were plotted at different physiological conditions i.e., at pH 2.2, pH 7.4, pH 9.4, saline and distilled water. The change in absorbance versus concentration was plotted to get standard curves in the respective media. Ten point calibration graphs were constructed covering a concentration range of 2–20 µg/mL of ondansetron. Three independent readings were obtained at each concentration. Linear relationships between the absorbance versus the concentration of ondansetron drug were observed. The value of the coefficient (R^2^) exceeded 0.99 and the standard deviations of the slope and intercept were low in each medium i.e., pH 2.2, pH 7.4, pH 9.4, saline and distilled water.

#### 3.3.2. Drug Loading Efficiency (DLE)

The DLE for SDS/FeSP nanocomposite was determined by loading the drug ondansetron onto the SDS/FeSP nanocomposite. The amount of drug loaded was observed by taking readings of the absorbance of supernatant solution left after the loading to find out the amount of leftover drug in the supernatant solution. The drug loading efficiency for SDS/FeSP nanocomposite was found to be 48.2%, as calculated by the Equation (1).

#### 3.3.3. Drug Release

**Figure 5:**
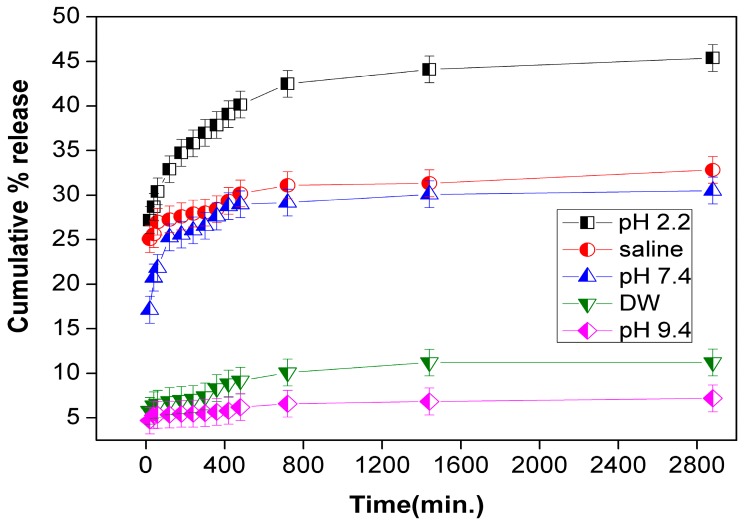
Cumulative % ondansetron release by SDS/FeSP nanocomposite.

The cumulative drug release of ondansetron was observed for the SDS/FeSP nanocomposite. The detailed results are shown in Figure 5. To study the cumulative drug release at different pH values for different time intervals, i.e., from 20 min to 48 h, a graph of cumulative release versus time was plotted. Release was determined by evaluating UV-visible spectral absorbance values as mentioned in Equation (2). The order of drug release observed in different media was pH 2.2 (45.38%) > saline (pH 5.5) (32.82%) > pH 7.4 (30.50%) > distilled water (DW) (11.22%) > pH 9.4 (7.20%). The maximum release of encapsulated drug was found to be about 45.38% at pH 2.2. As the pH of medium changes from acidic to basic, i.e., from 2.2 to 9.4 pH there is appreciable decrease in the drug release from 45.38% to 7.20% for the SDS/FeSP nanocomposite. It has thus been observed that ondansetron release from SDS/FeSP nanocomposite is pH-responsive and depends upon the respective pH of the medium.

**Figure 6:**
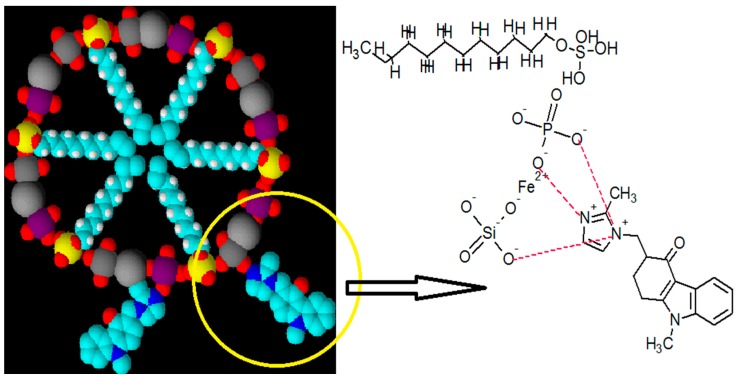
Possible mechanism of interaction of ondansetron SDS-FeSP nanocomposites.

The SDS/FeSP nanocomposite drug release depends upon two phenomena: (i) the exchange mechanism and (ii) the desorption mechanism. The poor performance at a basic pH is due to the destruction/interference of the SDS/FeSP in basic medium resulting in a desorption mechanism only. The exchange mechanism gets suppressed in basic medium due to the higher concentrations of anions, whereas at lower acidic pH both mechanisms, i.e., exchange and desorption, operate synergistically thus resulting in higher ondansetron release. The presence of higher levels of cations at lower pH facilitates the exchange mechanism. Ondansetron is a carbazole and contains two tertiary amino groups. Both of them are part of the heterocyclic ring. The methylated nitrogen is in conjugation with the electron withdrawing enone group and thus electrons are not present for protonation. Hence the presence of H^+^ ions in acidic medium helps in protonation of the tertiary nitrogen of diazole ring simultaneously, the phosphate/sulphate group present in SDS/FeSP, undergoing exchange and dissociation under a pH range of 0–3 resulting in a higher release of drug. The less drug release in DW is because in DW no ions are present thus the ion exchange mechanism does not operate in DW medium. The interpretation of the results predicts that the SDS/FeSP nanocomposite shows good controlled drug release performance. Moreover, all the release profiles of the SDS/FeSP nanocomposite exhibit a small burst release in the first 1 hour and then slow releases at different rates. The results suggest that it is possible to control the release rate of ondansetron from SDS/FeSP nanocomposite by adjusting the pH of the medium. The possible interaction of ondansetron with SDS/FeSP nanocomposite is shown in Figure 6.

#### 3.3.4. Mechanism of Drug Release

The mechanism of drug release was assessed by applying Fick’s equation [41,42]:

(5) $$\frac{M_{t}}{M_{\propto }}=Kt^{n}$$

where Mt and M∞ are the drug released at time “t” and equilibrium, respectively. K is a constant and n is the diffusion exponent of the release mechanism.

**Figure 7:**
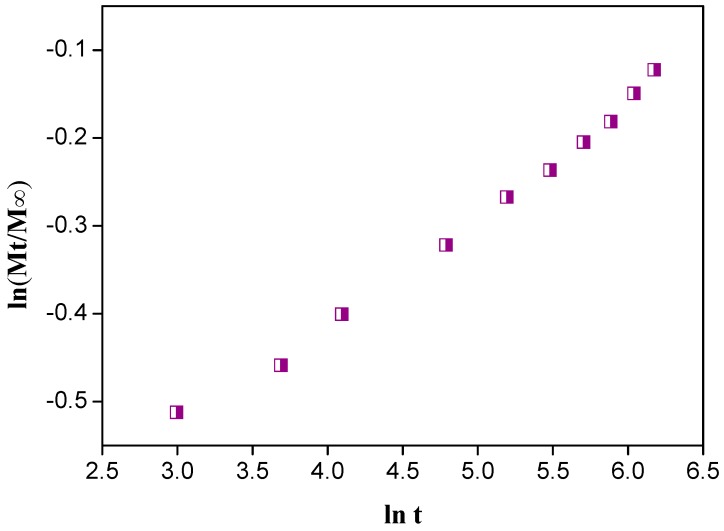
ln t versus ln(Mt/M∞) plot for Fick’s rule.

We get the values of k and n from the slope and intercept of a plot (Figure 7) between ln(Mt/M∞) and lnt [43]. The value of n was 0.8 with R^2^ value of 0.99, indicating a non-Fickian diffusion of the drug [42].

### 3.4. Cytotoxicity Studies

**Figure 8:**
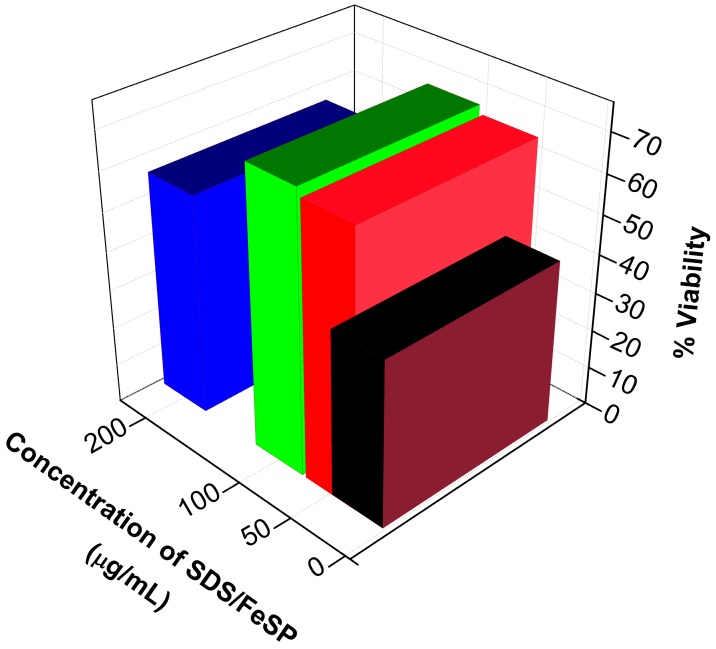
Viability effect with the increase in concentration of SDS/FeSP nanocomposites.

To reveal the cytotoxic effect [44] of SDS/FeSP nanocomposite, the viability of PBMCs was assayed by the MTT method.PBMCs were treated with SDS/FeSP nanocomposite at a range of concentrations from 25 to 200 µg/mL as shown in Figure 8. Our data revealed that SDS/FeSP nanocomposite is not toxic to human cells at any tested concentration. We also found enhanced cell viability from 25 to 150 µg/mL as compared to PBMCs treated with control (data has been normalized by control-treated PBMC as given in the protocol). The highest cell viability effect of SDS/FeSP nanocomposite was observed at 100 µg/mL, with 71% enhanced cell viability after 24 h of incubation (Figure 8).

## 4. Conclusions

The SDS/FeSP nanocomposite exhibited excellent properties for controlled drug delivery and has non-cytotoxic nature. The controlled release of the drug ondansetron has been explored in simulated GIT conditions. The synthesized SDS/FeSP nanocomposite showed good release of the drug. FTIR spectra confirmed the synthesis of the material by showing the diverse peaks of the different functional groups present in the SDS/FeSP nanocomposite. The results suggest that it is possible to control the release rate of ondansetron from SDS/FeSP nanocomposite by varying the pH of the medium. The SDS/FeSP nanocomposite had a proliferative effect with the increase in concentration, which clearly suggested that it regulates the immune response. Thus SDS/FeSP nanocomposite showed its paramount use as targeted controlled drug delivery system.
